# Experimental Study on Monitoring Equipment for the Scouring and Sedimentation of Wharf Bank Slopes Based on Heat Transfer Principles

**DOI:** 10.3390/s25051430

**Published:** 2025-02-26

**Authors:** Jilong Yin, Huaqing Zhang, Mengmeng Liu, Qian Ma

**Affiliations:** 1 College of River and Ocean Engineering, Chongqing Jiaotong University, Chongqing 400074, China; yinjilong121@163.com (J.Y.); tjzhq1@163.com (H.Z.); 2 National Engineering Research Center of Port Hydraulic Construction Technology, Tianjin Research Institute for Water Transport Engineering, Ministry of Transport, Tianjin 300456, China; 3 Institute of Marine Energy and Intelligent Construction, Tianjin University of Technology, Tianjin 300384, China; ma_qian@tju.edu.cn

**Keywords:** wharf bank slope, distributed fiber optic sensing, scouring and sedimentation, water–soil interface detection, temperature gradient, real-time monitoring

## Abstract

The scouring and sedimentation of wharf bank slopes significantly impact port safety and efficiency. To overcome the limitations of existing monitoring technologies in real-time capability, adaptability, and precision, this study introduces an innovative device based on distributed fiber optic sensing technology. By analyzing changes in the temperature gradient at the water–soil interface, the device enables dynamic monitoring of the results of scouring and sedimentation processes. It employs a modular design, integrating a linear heat source with fiber optic temperature sensing to capture high-resolution changes. Laboratory experiments evaluated variables such as heating duration, pipe material, pipe diameter, and fiber winding pitch. Results show optimal performance with a 20-min heating duration, with PVC sensors offering higher sensitivity and steel sensors providing greater stability. This study presents a high-precision, real-time solution for monitoring wharf bank slopes, offering insights for equipment optimization and engineering applications.

## 1. Introduction

With the rapid development of the global economy, international trade has become increasingly frequent. Water transportation, characterized by its low cost and high capacity, has emerged as an efficient and economical mode of transport [1]. As critical nodes in water transportation, wharves play an indispensable role in ensuring the smooth flow of logistics. The bank slope of a wharf, being its core structure, is essential for providing safe and reliable berthing conditions for ships [2,3]. However, due to the combined impacts of natural factors (e.g., seasonal seabed evolution causing flood-induced sedimentation and scouring [4,5]) and human activities (e.g., altered hydrodynamics due to wharf construction [6]), the scouring and sedimentation of bank slopes are frequent phenomena. These processes not only reduce port operational efficiency but can also lead to water quality deterioration and ecological damage [7].

In recent years, the study of the scouring and sedimentation of bank slopes has gained increasing attention. Driven by advancements in monitoring technologies, achieving real-time monitoring and dynamic assessment of these processes has become a hot topic in both academia and engineering [8]. The dynamic processes of scouring and sedimentation are complex, influenced by factors such as water flow velocity, wave variations, and sediment characteristics, which significantly increase the difficulty of monitoring. Traditional methods, while capable of providing basic information about bank slopes, still face limitations in terms of real-time capability, precision, and adaptability. Existing monitoring equipment spans various technologies, including acoustic side-scan sonar, multibeam bathymetric systems, acoustic Doppler current profilers (ADCP) [9,10,11], temperature, salinity, and turbidity sensors based on electrical signals [12], and optical crack detectors [13]. While these devices have their merits, traditional monitoring techniques exhibit notable drawbacks for the real-time monitoring of scouring and sedimentation. For example, sonar systems, though capable of providing accurate bathymetric data, are constrained by slow measurement speeds and response times, making it difficult to capture the dynamic scouring and sedimentation processes in real-time. Multibeam bathymetric systems and ADCP, though effective in measuring water flow and sediment distribution, are limited by measurement frequency and environmental conditions, restricting their use in long-term, high-frequency monitoring.

The monitoring of sedimentation processes, especially the identification of the water–soil interface, remains a technical challenge. Current methods include contact probing, soil pressure testing, optical depth sounding, sonar monitoring, laser depth sounding, and satellite remote sensing. Contact probing, a traditional mechanical measurement method, while simple to operate, suffers from low efficiency and accuracy and is only suitable for shallow water, failing to meet real-time demands in complex environments [14]. Soil pressure testing, which infers sediment thickness by measuring underwater soil pressure, is constrained by sensor precision and measurement location and depth [15]. Non-contact methods like laser and optical depth sounding offer certain levels of accuracy but are affected by water turbidity and exhibit significant errors in underwater monitoring [16]. Sonar monitoring, a widely used scouring and sedimentation technique, has limitations in measurement speed and precision and often requires specialized vessels for repeated traversals, diminishing its effectiveness in dynamic monitoring [17]. Satellite remote sensing, while advantageous for large-scale monitoring, lacks precision and is constrained by meteorological conditions, making it unsuitable for efficient real-time monitoring in complex marine environments [18]. Fiber optic sensing technology has already found significant applications in geohydrological monitoring [19]. For instance, Fiber Optic Distributed Temperature Sensing (DTS) technology can be used to measure soil temperature, thereby inferring changes in soil moisture content [20].

To address the shortcomings of existing monitoring technologies in terms of real-time capability and adaptability, this study proposes a monitoring device for wharf bank slope scouring and sedimentation based on distributed fiber optic sensing technology. Utilizing the significant thermal property differences at the water–soil interface, the device captures dynamic changes in the temperature gradient during heat transfer using fiber optic temperature sensing. The modular structure enhances adaptability and operational convenience, while the combination of a linear heat source and distributed fiber optic sensing technology achieves breakthroughs in high-resolution and real-time monitoring capability. Laboratory experiments systematically examined the feasibility and reliability of this technology, focusing on variables such as heating duration, pipe material, pipe diameter, and fiber winding pitch. Results demonstrate the device’s capability to accurately identify the water–soil interface’s position and dynamic evolution, with stable performance and strong adaptability. The outcomes not only provide valuable experience for the engineering application of distributed fiber optic sensing technology but also lay a solid foundation for optimizing equipment design and practical implementation.

## 2. Theoretical Foundations and Research Methods

### 2.1. Heat Transfer Theory of Water–Soil Interface Identification

Water and soil exhibit significant differences in their thermodynamic properties, leading to distinct characteristics in their heat transfer processes. Water has a higher specific heat capacity and thermal conductivity, enabling rapid heat transfer primarily through thermal convection. In contrast, soil has a lower specific heat capacity and thermal conductivity, with heat mainly propagating through thermal conduction at a slower rate. The temperature gradient at the interface is pronounced, resulting in an abrupt temperature change. This temperature anomaly at the interface provides a theoretical basis for identifying the water–soil interface.

**Figure 1:**
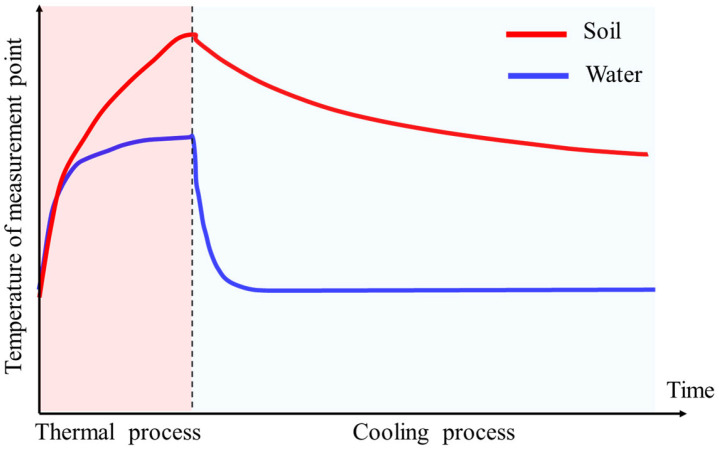
Schematic of heating and cooling with measurement points in water and soil media.

Figure 1 illustrates a typical schematic of heat conduction at the water–soil interface. During the heating process, the heat source operated at a constant power as a linear heat source. During the cooling process, the initial temperature of the heat source was set to the temperature at the end of the heating phase, followed by natural cooling with no additional heat input. As shown, when a linear heat source is applied to the water–soil boundary, the distinct thermal conductivities of water and soil result in significant differences in the speed and distribution characteristics of heat transfer in these two media. During heating, water rapidly transfers heat to its surroundings due to its higher thermal conductivity, whereas heat transfer in soil is slower due to its lower thermal conductivity. Over time, the temperature difference at the water–soil interface becomes increasingly apparent, eventually forming a pronounced temperature gradient. This abrupt temperature change at the water–soil interface provides a theoretical basis for its identification. By introducing a specific heat source (e.g., a linear heat source) at the water–soil interface, the differences in temperature conduction between water and soil can be utilized to precisely determine the interface’s location, thereby offering effective support for monitoring sedimentation and erosion.

### 2.2. Unsteady-State Heat Transfer Model Based on a Linear Heat Source

In the unsteady-state heat transfer model with a linear heat source, the heating power of the heat source remains constant over time. Due to the differences in thermal conductivity between water and soil, the rate of heat diffusion also varies. To effectively describe this process, based on the unsteady-state heat transfer Equation (1), the temperature change caused by the heat source can be expressed as follows [21]:

(1) $$\frac{\partial T}{\partial t}=\frac{\lambda }{\rho c}{𝛻}^{2}T+\frac{Q}{\rho c}$$

where λ is the thermal conductivity of the soil or water, ρ is the density, c is the specific heat capacity, *Q* is the heating power, and ${𝛻}^{2}T$ is the second spatial derivative of temperature. Due to the significant difference in thermal conductivity between water and soil, heat diffusion occurs at markedly different rates in the two media. Water exhibits faster temperature changes, while soil shows slower temperature variations, creating a distinct temperature gradient at the water–soil interface. This gradient serves as the theoretical foundation for identifying the interface. Specifically, the analytical solution for temperature change during heat source heating in water and soil are as follows:

**Heat source heating in water:** Assuming the heat source in water begins heating from the surface, the temperature distribution can be described by Equation (2):

(2) $$T\left(t\right)-T_{\infty }=\left\{\begin{array}{l} \frac{Q}{Ah}\left(1-\frac{\left|Q-Ah\left(T_{0}-T_{\infty }\right)\right|}{Ah}e^{-\frac{Ah}{\rho_{w}c_{w}V}t}\right),\;0<t<t_{1}\\ \left(T_{0}-T_{\infty }\right)e^{-\frac{hA}{\rho_{w}c_{w}V}\left(t-t_{1}\right)},\;\;\;\;\;\;\;\;\;\;\;\;\;\;t\geq t_{1} \end{array}\right.$$

where *Q* is the heating power; *h* is the convective heat transfer coefficient; *A* is the contact area between the unit length linear heat source and the seawater; *T*_0_ is the temperature of the surface of the linear heat source; *T*_∞_ is the initial temperature of the water medium; *ρ*_w_ is the density of the water medium; *c*_w_ is the specific heat capacity of the water medium; *V* is the volume of the contact section between the unit length linear heat source and the seawater; *t* is time.

**Heat source heating in soil:** For soil, the temperature changes more slowly, and the analytical solution is given by Equation (3):

(3) $$T\left(t\right)-T_{0}=\left\{\begin{array}{l} \frac{Q}{4\pi \lambda }\left(\ln\frac{4at}{r^{2}}-\gamma \right),0\leq t<t_{1}\\ \frac{Q}{4\pi \lambda }\ln\frac{t}{t-t_{1}},t>t_{1} \end{array}\right.$$

During the heating process, when *T*(*t*) = *T*_0_, the heat is transferred exactly to the position at a distance *r* from the linear heat source. After rearranging Equation (4), the relationship between Δ*t* and Δ*t* can be obtained as follows:

(4) $$\Delta x=\sqrt{\frac{4\alpha \Delta t}{e^{\gamma }}}$$

Since the linear heat source is considered infinitely long, physical quantities related to the length of the linear heat source are taken as unit length. Equation (3) is further transformed into the following:

(5) $$\Delta T_{s}=\frac{\Delta Q}{\rho_{s}c_{s}\pi (\left(r_{1}+\Delta x{)}^{2}-{r_{1}}^{2}\right)}$$

where *r*_0_ is the radius of the linear heat source, *r*_1_ is the inner radius of the tubular soil medium corresponding to each Δ*x*; and *ρ*_s_ is the density of the soil medium; *c*_s_ is the specific heat capacity of the soil medium.

By using the temperature variation models for the two media and combining the abrupt temperature change at the water–soil interface, the physical location of the water–soil interface can be accurately identified. In practical applications, monitoring equipment can dynamically track the evolution of the water–soil interface based on the gradient of this temperature change.

## 3. Monitoring Equipment Design and Development

### 3.1. Principles of Overall Equipment Design

**Figure 2:**
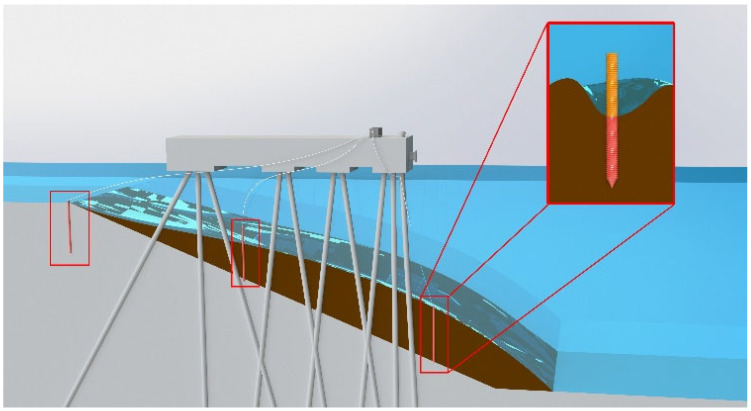
Schematic diagram of the principle of monitoring equipment for erosion and deposition in dock slopes.

To accurately capture the dynamic temperature changes in a linear heat source during heating or cooling processes in the water and soil of dock slope areas, and to identify in real time the position of the water–soil interface as well as its erosion and deposition states, this study designed an innovative monitoring device based on Distributed Fiber Optic Sensing (DFOS) technology. By incorporating intelligent and modular designs, the device simplifies the data acquisition and analysis process, significantly improving monitoring efficiency and accuracy. The design principle and deployment in practical applications are illustrated in Figure 2 [22].

**Figure 3:**
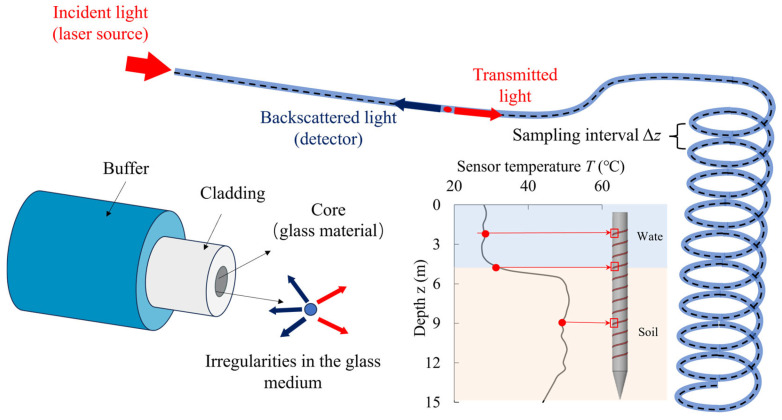
Schematic diagram related to the working principle of distributed fiber optics.

The device uses a linear heat source as a temperature disturbance source, introducing a controlled thermal variation field to create a significant temperature gradient at the water–soil interface. Distributed fiber optic sensors (DFOS) deployed along the linear heat source, leveraging their high-resolution and anti-interference capabilities, capture temperature signals in real time. By analyzing the dynamic changes in temperature over time and space, the device accurately identifies the real-time position and dynamic evolution of the water–soil interface. The working principle of DFOS is based on changes in the scattering signal within the optical fiber, enabling the distributed measurement of parameters such as strain, temperature, and acoustic signals along the entire length of a single optical cable. Typically, the optical cable consists of one or more optical fibers encapsulated within a protective sheath, allowing for high-precision measurements without the need for additional discrete sensors (e.g., fiber Bragg gratings or Fabry–Pérot interferometers), as shown in Figure 3.

The core advantage of Distributed Fiber Optic Sensing (DFOS) technology lies in its ability to perform continuous measurements along the entire length of the optical cable, providing high spatial resolution information on strain and temperature distribution. Among various DFOS techniques, this study selects Optical Frequency Domain Reflectometry (OFDR) based on Rayleigh scattering as the monitoring method. OFDR technology offers exceptional spatial resolution, enabling precise measurements at millimeter-scale intervals. In this study, the measuring point interval refers to the distance along the direction of the support rod, corresponding to the actually measured height of the interface. The measuring point interval is set to Δ*z* = 1 mm (as shown in Figure 3). The length of a measuring point refers to the length along the direction of the optical fiber, which is the result directly measured by the sensor. Each measuring point corresponds to a unit with a length of less than 1 cm. This technology allows for highly spatially distributed and in-depth monitoring over optical fiber paths several meters long. The distributed measurement of the Rayleigh scattering signals within the optical fiber can be described by Equation (6):

(6) $$P_{R}\left(z\right)=P_{0}\cdot e^{-2\alpha z}\cdot \eta \left(z\right)$$

where $P_{R}\left(z\right)$ represents the scattered power at a distance *z*, $P_{0}$ is the incident light power, *α* is the fiber attenuation coefficient, and $\eta \left(z\right)$ represents the change in the scattering coefficient caused by temperature or strain. The fiber demodulation system analyzes these changes to generate high-resolution temperature distribution curves, thereby achieving precise monitoring of the water–soil interface position and its dynamic evolution.

This device, by integrating linear heat sources with distributed fiber optic sensing technology, provides an innovative solution for monitoring erosion and deposition in complex slope environments. Its high resolution, continuous distributed measurement, and modular design offer superior performance in accuracy, real-time monitoring, and adaptability compared to traditional methods.

### 3.2. Modular Design of the Equipment

**Figure 4:**
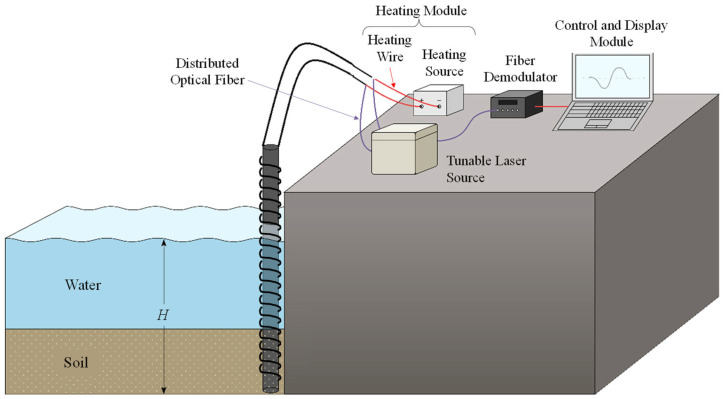
Design principle schematic of dock shore slope erosion and sedimentation monitoring equipment.

Based on the principles outlined above, the equipment adopts a modular design, as shown in Figure 4. It is divided into a sensing module, a heating module, and a control and display module to ensure clear functionality, efficient operation, and adaptability and stability in complex environments.

The sensing module is the core component of the equipment, responsible for collecting temperature data at the water–soil interface. The key components of this module include distributed fiber Bragg grating temperature sensors and a linear heat source. The fiber optic sensors and heat source are arranged helically along the supporting structure to capture subtle changes in the temperature gradient. The exterior of the sensors is coated with corrosion-resistant carbon fiber composite material and epoxy resin, enhancing their durability in marine environments while effectively reducing heat loss between the optical fiber and the external medium.

The design of the sensing module also emphasizes installation convenience, allowing it to adapt to various water depths and geological conditions.

The heating module, as the power source for heat input, provides stable thermal disturbances to the monitored area through the linear heat source. The design of this module focuses on the flexibility of power adjustment, enabling the equipment to adaptively regulate the rate of temperature change in different medium environments, thereby optimizing the temperature gradient at the water–soil interface. To reduce energy consumption, the module uses highly efficient heating materials and incorporates thermal insulation measures in its structural design, further enhancing the system’s energy efficiency.

The control and display module manages the automated operation and data processing of the equipment. It integrates a temperature demodulator, an automatic control system, and a graphical display unit. The temperature demodulator analyzes the scattered signals collected by the sensors to plot real-time temperature distribution curves within the monitored area, providing intuitive data support for subsequent analysis. The automatic control system dynamically adjusts the operating parameters of the equipment and promptly issues alarms in case of anomalies in the sensors or heating module.

Additionally, the module is equipped with long-term data storage and processing capabilities, providing a robust data foundation for extended monitoring and trend analysis.

The modular design of the equipment makes the installation process simpler and allows for quick adaptation to different field environments. In order to evaluate the performance of the equipment modules designed in this article, especially the sensor module, in different water and soil media, system tests will be conducted in a controlled environment to further verify its effectiveness and accuracy in practical applications. These tests will ensure that the equipment can operate stably and accurately in actual shore slope monitoring, supporting the monitoring and analysis of relevant erosion and sedimentation processes.

## 4. Experimental Verification and Result Analysis

### 4.1. Experimental Setup

To evaluate the water–soil interface identification capability of the monitoring equipment under different conditions, this study designed a systematic indoor validation experiment. In the experiment, the initial temperatures of the water, soil, and heat source were set to be the same as the room temperature, at 18 °C. The experiment is based on port engineering structure testing facilities to simulate actual shore slope erosion and sedimentation environments, exploring the impact of multiple variables on equipment performance. The experimental design focuses on the heat transfer characteristics of linear heat sources, with external heating durations set at 10 min, 20 min, 30 min, and 40 min. The study primarily analyzes the effects of three variables on monitoring performance: pipe material, pipe diameter, and optical fiber winding pitch. Two pipe materials, steel and PVC, are selected, with pipe diameters set at 2 cm, 5 cm, and 10 cm. The optical fiber winding pitches are set at 1 cm, 2 cm, and 4 cm. To enhance the sensor’s anti-interference ability and corrosion resistance, the sensor surface is coated with epoxy resin or carbon fiber + epoxy resin. Based on the above variable combinations, 18 prototypes were designed and produced, with specific parameters as shown in Table 1. Except for the variables listed in the table, all other variables, including sensor material, fiber winding pitch, and heat source power, were kept consistent across experiments. The purpose of designing these prototypes is to comprehensively address environmental conditions in actual applications, providing a scientific foundation for the comprehensive evaluation of the equipment’s performance.

### 4.2. Equipment and Materials

**Figure 5:**
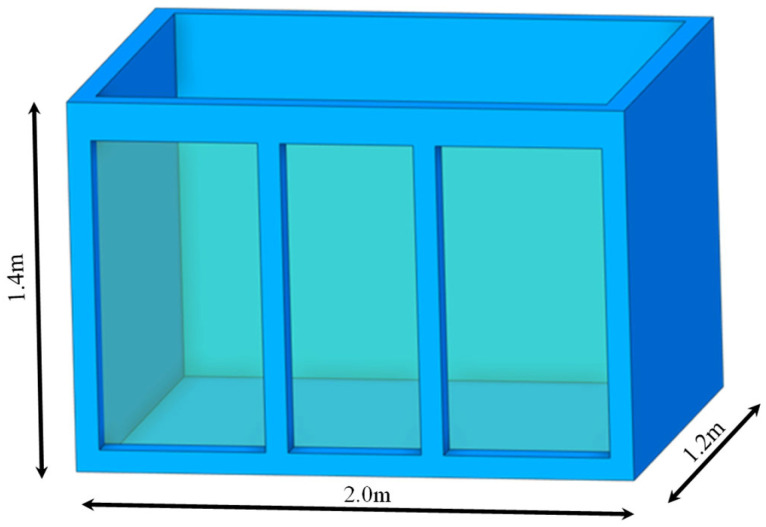
Schematic diagram of the test tank.

The experiment is conducted in a custom-designed dual-slot testing device, with each test slot having internal dimensions of 2 m × 1.2 m × 1.4 m. The tank structure is made of steel and double-layer glass, ensuring both sealing and visibility (as shown in Figure 5). The experimental soil is kaolin, with a soil-to-water ratio of 1:1, mixed thoroughly to create a slurry. To prevent water leakage, a layer of PVC mesh grid covered with geotextile is laid at the bottom of the tank, followed by a layer of gravel and then the soil fill. After the filling is completed, natural drainage is allowed until there is no standing water on the surface. The sensors are then placed, and the water depth is adjusted to 1 m to simulate the water–soil interface environment.

**Figure 6:**
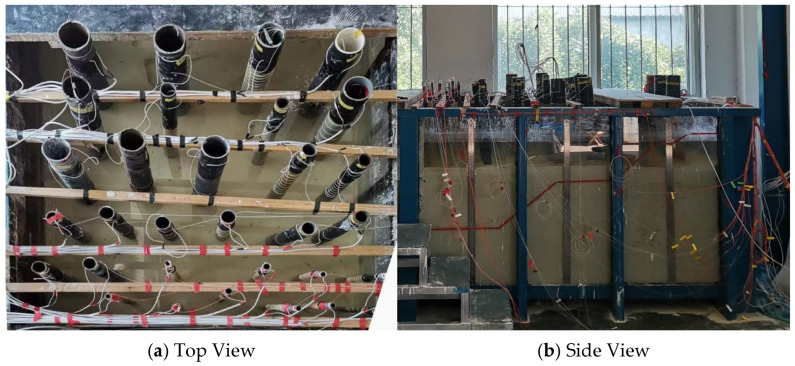
Layout of rods and sensor placement.

During the sensor placement, the test tank is divided into multiple zones based on the pipe diameter, with sensors of the same specifications arranged in each zone. The pipes are arranged from smaller diameters to larger ones to prevent interference during subsequent placement. To minimize interference between sensors, the spacing between the rods is kept at approximately 20 cm. The optical fibers and heating cables are neatly tied together and fixed onto wooden rods (as shown in Figure 6). This arrangement not only facilitates unified management of the sensors but also ensures the stability of the connections and the reliability of the measurements.

### 4.3. Procedure and Operations

The experimental process consists of four main stages: equipment debugging, parameter setting, active heating monitoring, and data recording. First, before starting the experiment, a comprehensive inspection and debugging of all equipment is carried out to ensure that components such as the power supply, relays, temperature measuring instruments, and channel generators are functioning properly. The water level in the test tank is then adjusted to the design height, and the sensor coating is checked for integrity. If any damage is found, it must be replaced promptly. Next, parameters are set according to the experimental plan, including the heating power, heating duration of the linear heat source, and test channel configurations, to ensure the experimental conditions align with the design requirements. During the experiment, the linear heat source system is activated, and heating is carried out under the preset conditions. The temperature measurement system continuously collects data from each sensor’s measurement point and automatically generates a temperature distribution curve. If any fiber optic cable is broken or if there is an abnormal temperature, the system will trigger an alarm and log the fault location. If the temperature at any measurement point exceeds the safety threshold, the experiment will be halted based on the equipment’s condition. At the end of the experiment, all recorded data are exported to a designated folder and archived with the experimental number. This standardized operational procedure ensures the reliability and repeatability of the experiment and provides comprehensive data support for subsequent data analysis and result discussion.

### 4.4. Results Analysis and Evaluation

#### 4.4.1. Analysis and Evaluation Methods

When analyzing the impact of various variables on the sensor’s monitoring performance, the evaluation is primarily conducted from two aspects: differentiation evaluation, which refers to the differences in monitoring results across different media, and stability evaluation, which focuses on the consistency of monitoring results within the same medium.

Differentiation evaluation is performed by calculating the difference in the average temperature increment $\Delta \bar{T}$ across different media and the temperature gradient $\Delta {\bar{T}}_{s-w}$ near the water–soil interface, among other indicators. This helps distinguish the sensor’s response differences in various media, especially near the water–soil interface. It provides the basis for identifying the water–soil interface, ensuring that the sensor can accurately locate the interface between different media, and subsequently determine the sedimentation thickness. The expression for the average temperature increment $\Delta \bar{T}$ is given by Equation (7):

(7) $$\Delta \bar{T}=\frac{\Delta T_{1}+\Delta T_{2}+\cdots \cdots +\Delta T_{n}}{n}$$

where $\Delta T_{1}$, $\Delta T_{2}$ …… $\Delta T_{n}$ represent the temperature increments at the measurement points within the same medium. The value of n can be set according to the requirements of the actual engineering application. In this study, the temperature averaging range is 0.1 m.

The expression for the temperature gradient $\Delta {\bar{T}}_{s-w}$ is given by Equation (8):

(8) $$\Delta {\bar{T}}_{s-w}=\Delta {\bar{T}}_{s}-\Delta \bar{T_{w}}$$

where $\Delta \bar{T_{s}}$, $\Delta \bar{T_{w}}$ represent the average temperature increments at the measurement points within 0.1 m above and below the water–soil interface in the soil and water, respectively, and $\Delta {\bar{T}}_{s-w}$ represents the temperature gradient.

Stability evaluation is conducted using indicators such as the standard deviation of temperature increment σ and the uniformity index UI to measure the stability and uniformity of the sensor’s monitoring results within the same medium. This ensures that the monitoring curve does not exhibit significant fluctuations in the same medium, thereby guaranteeing that the identification of sediment thickness is not subject to unnecessary interference.

The expression for the standard deviation of the temperature increment σ is given by Equation (9):

(9) $$\sigma =\sqrt{\frac{\sum_{i=1}^{n}{\left(T_{i}-\bar{T}\right)}^{2}}{n}}$$

The expression for the uniformity index UI is given by Equation (10):

(10) $$UI=\frac{1}{\bar{T}}\sqrt{\frac{\sum_{i=1}^{n}{\left(T_{i}-\bar{T}\right)}^{2}}{n}}$$

where $T_{i}$ represents the temperature increment at each measurement point, $T_{i}$ is the average temperature increment, and n is the number of measurement points.

Through multiple experiments, the effects of three variables—sensor rod material, pipe diameter, and winding pitch—on sensor performance are analyzed. Sensitivity analysis is conducted to study the impact of each individual variable on sensor performance. By changing a single parameter and observing the resulting changes in monitoring outcomes, the influence of each parameter on sensor performance can be further understood, enabling the optimization of sensor design parameters. Through qualitative and quantitative analysis of the differentiation and stability of monitoring results, the sensor’s ability to accurately locate the interface in different media is ensured. At the same time, the stability and reliability of the sensor in the same medium are evaluated, providing a comprehensive reflection of the sensor’s performance. Based on these findings, the design parameters of the sensor can be optimized to achieve optimal monitoring performance.

#### 4.4.2. Sensor Performance Analysis by Material

**Figure 7:**
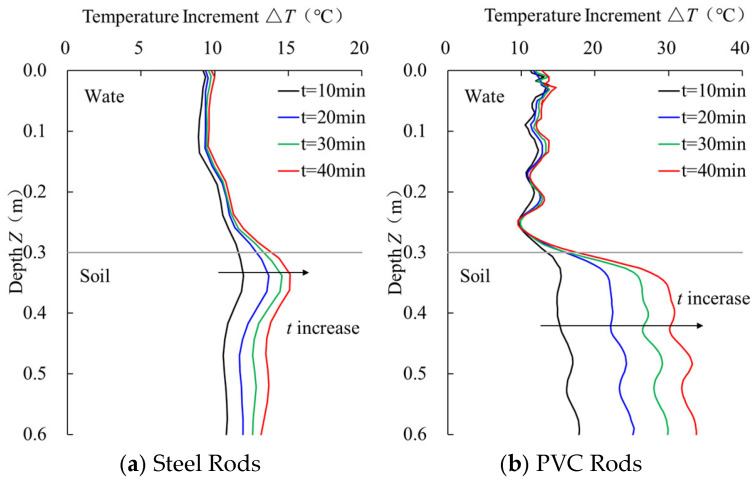
Temperature monitoring curves of sensor rods with different materials.

Under heating durations of 10 min, 20 min, 30 min, and 40 min, the temperature monitoring curves of steel and PVC sensors are shown in Figure 7. The corresponding differentiation and stability evaluation results are listed in Table 2 and Table 3, respectively.

As shown in Figure 7, the temperature increment of the steel sensor in the water increases linearly with the heating time, with average temperature increments of 9.55 °C, 9.86 °C, 10.00 °C, and 10.17 °C, showing a gradual slowing of the increase. In the soil, the temperature increment increases significantly during the 10–20 min heating period, and the curve stabilizes after 20 min of heating. In contrast, the PVC sensor’s temperature monitoring results show higher sensitivity, with the temperature increment in the water ranging from 11.42 °C to 12.46 °C, and the temperature increment in the soil is significantly higher than that of the steel sensor.

From the temperature gradient data in Table 2, the PVC sensor exhibits a significantly higher temperature gradient at the water–soil interface compared to the steel sensor, demonstrating better interface identification capability. On the other hand, the stability evaluation results in Table 3 indicate that the steel sensor has a lower standard deviation in temperature difference and a higher uniformity index than the PVC sensor, indicating better stability.

#### 4.4.3. Sensor Performance Analysis by Pipe Diameters

**Figure 8:**
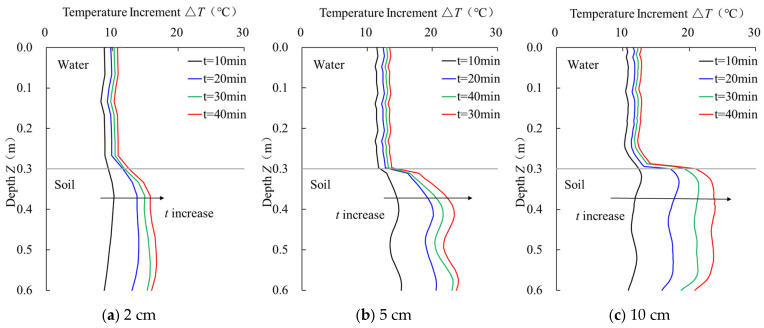
Temperature monitoring curves of sensors with different pipe diameters.

The temperature monitoring curves of sensor rods with different pipe diameters under varying heating durations are compared in Figure 8. The differentiation and stability evaluation data of the temperature monitoring curves for sensors with three different pipe diameters under different heating durations are shown in Table 4 and Table 5. As shown in Figure 8 and Table 4, the average temperature difference increments in both soil and water segments for sensors with 2 cm, 5 cm, and 10 cm diameters increase with the heating time. The most significant increase occurs between 10–20 min. For instance, the average temperature difference increments for the 10 cm diameter sensor increase by 5.66 °C and 1.79 °C in the soil and water segments, respectively, with an increase of 48.21% and 16.84%, indicating that the sensor’s sensitivity to heating duration improves as the heating time increases. Notably, for the 2 cm diameter sensor, the temperature gradient turns negative after 10 min of heating. This could be attributed to the sparse distribution of fiber optic measurement points on the 2 cm diameter sensor, making it difficult to accurately measure the temperature distribution on the sensor. In shorter heating times, there is less heat accumulation in the soil, and the heat exchange rate between the soil and water is greater than the heating rate of the linear heat source, causing the temperature increment in the soil to be smaller than that in the water. From Table 5, it can be observed that for sensors with all three pipe diameters, the standard deviation of the temperature differences in both soil and water segments gradually increases with the heating time, indicating that temperature fluctuations increase with longer heating durations. The temperature difference uniformity remains relatively unchanged across different heating durations, suggesting that the temperature distribution is relatively uniform. Among sensors with different pipe diameters, the temperature fluctuation generally increases as the heating time extends, indicating that longer heating durations have a negative impact on the sensor’s stability. The optimal heating duration should balance improved sensitivity with stability to ensure the best monitoring performance.

#### 4.4.4. Sensor Performance Analysis by Wrapping Pitches

**Figure 9:**
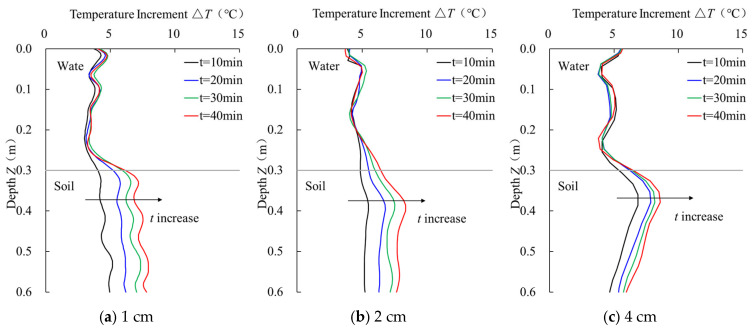
Temperature curves of sensors with different winding pitches.

The temperature monitoring curves of sensor rods with different fiber optic wrapping pitches under varying heating durations are compared in Figure 9. The differentiation and stability evaluation data of the temperature monitoring curves for sensors with two different wrapping pitches under different heating durations are shown in Table 6 and Table 7. As shown in Figure 9, the temperature curves of sensors with three different wrapping pitches in the water segment exhibit a high degree of overlap under different heating durations. According to Table 6, when the fiber optic wrapping pitch is 1 cm, the temperature gradients in water and soil media for heating durations of 10, 20, 30, and 40 min are 1.26 °C, 2.25 °C, 2.96 °C, and 3.72 °C, respectively. The greatest increase occurs between 10–20 min, with a rise of 78.57%. The temperature gradient increases by 31.56% and 25.68% in the next two durations, which is similar to the temperature gradient increase for sensors with wrapping pitches of 2 cm and 4 cm. Combining the temperature change data for sensors with three different wrapping pitches under various heating durations, it can be observed that the temperature change in the sensor rods becomes most significant after 20 min of heating. Furthermore, the degree of impact by the heating duration on sensors with different wrapping pitches is quite similar.

Analyzing Table 6 and Table 7, the average temperature increment differences for sensors with wrapping pitches of 1 cm, 2 cm, and 4 cm are as follows: the sensor with a 1 cm pitch shows an increase in average temperature increment from 4.61 °C to 7.43 °C under heating durations of 10 to 40 min; the sensor with a 2 cm pitch increases from 5.33 °C to 7.93 °C; and the sensor with a 4 cm pitch increases from 6.33 °C to 7.98 °C. In the water segment, the average temperature increment for the 1 cm pitch sensor remains relatively low at all heating durations, with a maximum value not exceeding 3.71 °C. For the 2 cm pitch sensor, the average temperature increment in the water segment gradually increases, with a maximum value of 4.28 °C. The 4 cm pitch sensor maintains a relatively stable temperature increment in the water segment, reaching a maximum value of 5.04 °C. As for the temperature gradient, the 1 cm pitch sensor’s gradient gradually increases over different heating durations, with a maximum value of 3.72 °C; the 2 cm pitch sensor follows a similar pattern, reaching a maximum of 3.65 °C; and the 4 cm pitch sensor experiences smaller changes, with a maximum gradient of 2.94 °C. Comprehensive analysis reveals that the 1 cm pitch sensor performs relatively stably in the water segment but has smaller temperature increments in the soil segment, which reduces its sensitivity. The 2 cm pitch sensor strikes a balance, performing well in both temperature increments and sensitivity, though it has higher temperature increments in the water segment. The 4 cm pitch sensor shows stable temperature increments in the water segment while exhibiting relatively higher temperature increments in the soil segment, along with a lower temperature gradient. This analysis indicates that the fiber optic wrapping pitch significantly influences the sensor’s sensitivity and stability in different environments.

## 5. Conclusions

This article addresses the dynamic monitoring needs of dock shore scouring and sedimentation processes, designing and verifying an efficient monitoring device based on distributed optical fiber sensing technology. Through experiments, the monitoring performance of the device under multivariable conditions was evaluated, and the following main conclusions were obtained:

(1) The device uses a linear heat source as the temperature disturbance source, combined with a distributed optical fiber sensor, achieving real-time monitoring of the temperature gradient at the water–soil interface. Its modular design significantly improves the device’s adaptability and ease of operation in complex experimental environments. Experimental results prove that the device can accurately capture dynamic changes at the water–soil interface, meeting the needs for monitoring shore scouring and sedimentation.
(2) Experimental results indicate that a 20-min heating duration is the optimal operating point for the device’s performance, as it strikes a balance between temperature response sensitivity and monitoring data stability. PVC sensors perform excellently in responding to the temperature gradient at the water–soil interface, making them particularly suitable for monitoring scenarios that require high sensitivity. On the other hand, steel sensors demonstrate higher temperature distribution uniformity and stability under the same conditions, making them suitable for engineering environments requiring long-term monitoring.
(3) Pipe diameter has a significant impact on the device’s sensitivity and stability: sensors with smaller diameters are more sensitive to temperature changes but show slightly reduced stability when the heating time is short. Sensors with larger diameters exhibit more stable temperature distribution during long-term heating. The fiber optic winding pitch has little effect on the temperature difference in the water section, but it makes a significant contribution to the temperature difference increment and temperature gradient in the soil section. By adjusting the pitch, the device’s response in different media can be further optimized.

The findings of this study have profound implications for the future application of the proposed monitoring device. By enabling the high-precision, real-time monitoring of scouring and sedimentation processes, the device can significantly enhance the safety and operational efficiency of port facilities. Its adaptability to various environmental conditions, such as open water and complex terrains, makes it a versatile tool for coastal and riverbank monitoring. Furthermore, the insights gained from material selection (e.g., PVC for sensitivity, steel for stability) and parameter optimization (e.g., heating duration, pipe diameter, and winding pitch) provide a roadmap for tailoring the device to specific engineering needs. This technology has the potential to revolutionize infrastructure monitoring, not only in ports but also in other critical areas such as dams, bridges, and offshore platforms, where real-time data on sediment dynamics are crucial for structural integrity and disaster prevention. Future work will focus on scaling up the device for field applications and integrating it with advanced data analytics for predictive maintenance and risk assessment.

## Figures and Tables

**Table 1:** Summary of sensor prototypes.

Sensor Prototype Serial Number	Material	Pipe Diameter/cm	Winding Pitch/cm
1	Steel	10	4
2	Steel	5	4
3	Steel	2	4
4	Steel	10	2
5	Steel	5	2
6	Steel	2	2
7	Steel	10	1
8	Steel	5	1
9	Steel	2	1
10	PVC	9	1
11	PVC	9	4
12	PVC	9	2
13	PVC	5	4
14	PVC	5	2
15	PVC	5	1
16	PVC	2	4
17	PVC	2	2
18	PVC	2	1

**Table 2:** Temperature gradient of sensor rods with different materials.

Material	Heating Duration (min)	Average Temperature Difference Increment in Soil (°C)	Average Temperature Difference Increment in Water (°C)	Temperature Gradient (°C)
Steel	10	11.01	9.55	1.47
	20	12.29	9.86	2.44
	30	13.13	10.00	3.13
	40	13.90	10.17	3.73
PVC	10	16.18	11.42	4.76
	20	23.34	11.97	11.37
	30	28.04	12.22	15.81
	40	31.66	12.46	19.19

**Table 3:** Stability evaluation of temperature monitoring curves for sensor rods with different materials.

Material	Heating Duration (min)	Standard Deviation of Temperature Difference in Soil	Uniformity of Temperature Difference in Soil	Standard Deviation of Temperature Difference in Water	Uniformity of Temperature Difference in Water
Steel	10	0.471	0.043	0.744	0.078
	20	0.739	0.060	0.811	0.082
	30	0.755	0.057	0.774	0.077
	40	0.675	0.049	0.812	0.080
PVC	10	0.995	0.062	0.899	0.079
	20	1.082	0.046	1.063	0.089
	30	1.232	0.044	1.075	0.088
	40	1.390	0.044	1.301	0.104

**Table 4:** Temperature differentiation evaluation of sensor rods with different pipe diameters.

Pipe Diameter	Heating Duration (min)	Average Temperature Difference Increment in Soil (°C)	Average Temperature Difference Increment in Water (°C)	Temperature Gradient (°C)
10	10	11.74	10.63	1.11
	20	17.40	12.42	4.98
	30	21.00	12.79	8.21
	40	23.30	13.01	10.30
5	10	14.39	11.88	2.51
	20	19.81	13.85	5.96
	30	22.93	14.38	8.55
	40	21.63	13.60	8.03
2	10	9.66	10.44	−0.78
	20	13.65	11.71	1.94
	30	15.18	11.90	3.28
	40	16.07	11.91	4.16

**Table 5:** Stability evaluation of temperature monitoring curves for sensor rods with different pipe diameters.

Pipe Diameter	Heating Duration (min)	Standard Deviation of Temperature Difference in Soil	Uniformity of Temperature Difference in Soil	Standard Deviation of Temperature Difference in Water	Uniformity of Temperature Difference in Water
10	10	0.421	0.036	0.488	0.046
	20	0.525	0.030	0.938	0.076
	30	0.459	0.022	1.036	0.081
	40	0.697	0.030	1.246	0.096
5	10	0.632	0.044	0.964	0.081
	20	0.575	0.029	1.427	0.103
	30	0.773	0.034	1.578	0.110
	40	0.958	0.044	1.279	0.094
2	10	0.500	0.052	1.227	0.118
	20	0.394	0.029	1.364	0.117
	30	0.562	0.037	1.431	0.120
	40	0.619	0.038	1.224	0.103

**Table 6:** Temperature curves of sensor rods with different winding pitches.

Pitch (cm)	Heating Duration (min)	Average Temperature Difference Increment in Soil (°C)	Average Temperature Difference Increment in Water (°C)	Temperature Gradient (°C)
1	10	4.61	3.35	1.26
	20	5.91	3.66	2.25
	30	6.78	3.83	2.96
	40	7.43	3.71	3.72
2	10	5.33	4.29	1.04
	20	6.54	4.41	2.13
	30	7.12	4.48	2.64
	40	7.93	4.28	3.65
4	10	6.33	4.99	1.34
	20	7.29	4.92	2.37
	30	7.63	4.95	2.68
	40	7.98	5.04	2.94

**Table 7:** Stability evaluation of temperature monitoring curves for sensor rods with different winding pitches.

Pitch (cm)	Heating Duration (min)	Standard Deviation of Temperature Difference in Soil	Uniformity of Temperature Difference in Soil	Standard Deviation of Temperature Difference in Water	Uniformity of Temperature Difference in Water
1	10	0.332	0.072	0.401	0.120
	20	0.215	0.036	0.455	0.124
	30	0.344	0.051	0.466	0.122
	40	0.343	0.046	0.473	0.127
2	10	0.103	0.019	0.523	0.122
	20	0.164	0.025	0.496	0.112
	30	0.246	0.035	0.566	0.126
	40	0.270	0.034	0.549	0.128
4	10	0.486	0.077	0.795	0.159
	20	0.520	0.071	0.992	0.202
	30	0.475	0.062	0.987	0.199
	40	0.523	0.066	1.048	0.208

## Data Availability

The data presented in this study are available upon request from the corresponding author. The data are not publicly available due to the fact that the dataset production is part of the innovation point of this paper, so the dataset will not initially be open source; we will continue to explore new possibilities in the subsequent experimental research, and then the dataset will be made public along with the related code.
